# Sustainable Production and In Vitro Giardicidal Activity of Bioactive Compounds Derived from *Arthrospira Platensis*

**DOI:** 10.1007/s11686-026-01313-1

**Published:** 2026-06-06

**Authors:** Ketyline Lira de Lima, Júlia Sofia Neves de Sousa, Karina Evelyn de Lima Albuquerque Santos, Maria Rafaele Oliveira Bezerra da Silva, Raquel Pedrosa Bezerra, Romero Marcos Pedrosa Brandão Costa, Kethylen Barbara Barbosa Cardoso, Patrícia Melo Ferreira, Genésio Florentino Duarte Neto, Silvana de Fátima Ferreira, Daniela de Araújo Viana Marques

**Affiliations:** 1 Integrated Laboratory in Applied Biotechnology, Institute of Biological Sciences, University of Pernambuco, Santo Amaro, Recife, Pernambuco, 50100-130 Brazil; 2 Department of Animal Morphology and Physiology, Federal Rural of University of Pernambuco, Recife, Pernambuco, 52171-900 Brazil; 3 Laboratory of Biotechnology Applied to Infectious and Parasitic Diseases, University of Pernambuco, Santo Amaro, Recife, Pernambuco, 50100-130 Brazil

**Keywords:** Antiparasitic, Natural therapy, Microalgal bioactives, Phycochemicals, Drug-resistant parasites

## Abstract

**Background:**

Giardiasis remains a globally prevalent neglected parasitic disease, and limitations associated with conventional therapies highlight the need for alternative and complementary strategies. In this context, microalgae biocompounds have emerged as promising sources of antiparasitic agents.

**Objective:**

This study aims to evaluate the *in vitro* preliminary screening activity (vital dye exclusion).

**Methods:**

Aqueous extracts of *Arthrospira platensis* obtained by different extraction methods, as well as the bioactivity of the product derived from *in vitro* digestion (PDV), aiming to assess the influence of extraction and digestive bioactivation on antiparasitic efficacy. Extracts were produced by magnetic stirring and sonication, followed by exposure of *Giardia duodenalis* cysts to increasing concentrations, with metronidazole used as a reference drug. The PDV was generated using a standardized simulated gastrointestinal digestion model. Sonicated extracts showed enhanced membrane damage compared to mechanically stirred extracts, consistent with improved release of intracellular bioactive compounds. The IC_50_ values for the sonicated, agitated, and PDV samples were, respectively: 95,4; 153.1 and 43.6 μg/mL.

**Results:**

The PDV demonstrated activity across multiple concentrations and, under comparable conditions, outperformed metronidazole. These findings suggest that digestive processing may increase the bioaccessibility of active moieties responsible for antiparasitic effects.

**Conclusions:**

Overall, the results support *A. platensis* as a sustainable source of giardicidal biocompounds and highlight the relevance of physiologically relevant in vitro digestion models for predicting functional antiparasitic activity and guiding future translational research.

## Introduction

Giardiasis, caused by the protozoan *Giardia duodenalis* (syn. *G. lamblia*, *G. intestinalis*), remains a globally relevant waterborne disease, particularly in regions with limited sanitation infrastructure. Epidemiological data indicate that *Giardia duodenalis* infection affects about 2–5% of the population in high-income (developed) countries, whereas in low- and middle-income (developing) regions the prevalence can reach approximately 20–30%, particularly in settings with limited access to safe water and sanitation facilities [1]. Although improvements in sanitary control have reduced incidence in industrialized countries, giardiasis is still considered an emerging infection due to increased international travel and recurrent water contamination events [2].

Conventional pharmacological treatment relies mainly on nitroimidazole derivatives, such as metronidazole and tinidazole, which are effective but frequently associated with adverse effects, including gastrointestinal disturbances, neurological symptoms, and reports of mutagenic and carcinogenic potential [3, 4]. Moreover, increasing cases of therapeutic failure and parasite resistance have been documented, highlighting the urgent need for alternative antiparasitic strategies with improved safety profiles and sustainable production routes [5, 6].

In this scenario, the development of sustainable bioprocesses for the production of bioactive compounds has emerged as a promising approach in pharmaceutical and environmental biotechnology. Natural compounds obtained from photosynthetic microorganisms, particularly microalgae and cyanobacteria, have gained attention due to their chemical diversity, renewable biomass production, and low environmental impact [7, 8]. Among these organisms, the cyanobacterium *Arthrospira platensis* stands out as a well-established industrial platform, widely explored in food, nutraceutical, and pharmaceutical sectors [9]. Its biomass is characterized by high protein content (approximately 60% dry weight), elevated digestibility, and the absence of rigid cellulose-based cell walls, which favors downstream processing and extraction efficiency [10].

Bioactive compounds derived from *A. platensis* have been associated with antioxidant, anti-inflammatory, antitumor, and immunomodulatory activities [11]. However, despite its broad biotechnological potential, the application of *A. platensis* as a sustainable source of antiparasitic compounds, particularly targeting the environmentally resistant cystic form of *G. duodenalis*, remains insufficiently explored. Since cyst viability is a critical factor for transmission, persistence in aquatic environments, and outbreak dynamics, strategies aimed at reducing cyst survival are highly relevant from both prophylactic and therapeutic perspectives [12].

From a process engineering standpoint, the integration of green extraction techniques, efficient separation systems, and biologically relevant in vitro models is essential to advance sustainable pharmaceutical processes. Aqueous two-phase systems (ATPS), such as polyethylene glycol/phosphate systems, represent an environmentally friendly and scalable alternative for parasite purification and bioactivity assessment, minimizing the use of organic solvents and harsh conditions [13]. Additionally, in vitro digestion models can simulate gastrointestinal conditions, providing insights into the stability and bioactivity of compounds after oral administration.

Therefore, the present study proposes a sustainable approach to evaluate the membrane damage of Giardia cysts detected by eosin exclusion of biocompounds derived from *Arthrospira platensis*. The work investigates (i) the *in vitro* antiparasitic activity of aqueous extracts obtained from *A. platensis*, and (ii) the giardicidal potential of the *in vitro* digestion product (PDV) of the biomass. By integrating sustainable production, green separation processes, and biological evaluation, this study contributes to the development of environmentally responsible antiparasitic strategies.

## Materials and Methods

### Cultivation of Microorganisms

The *Arthrospira platensis* was obtained from the University of Texas Culture Collection (UTEX, 2010). The cyanobacterium was cultivated in a standardized culture medium, as previously described by Schlosser [14]. Cultivation was initiated with an initial biomass concentration of 50 mg/L and maintained under controlled conditions until the exponential growth phase. At the end of the exponential phase, the biomass was harvested by centrifugation at 10,000 rpm for 10 min at 4 °C. The recovered cells were subsequently freeze-dried and stored under appropriate conditions until further processing. All reagents employed throughout the experimental procedures were purchased from Sigma-Aldrich (St. Louis, MO, USA), unless otherwise specified.

### Preparation of Aqueous Extracts

Aqueous extracts were obtained by resuspending 0.3 g of freeze-dried *A. platensis* biomass in 3 mL of 0.02 M phosphate buffer (pH 7.0). Two extraction strategies were employed to evaluate the influence of processing conditions on extract composition: (i) sonication, consisting of 10 pulses of 1 min each, and (ii) magnetic stirring for 12 h at 4 °C, following the methodology described by Dinh, Hori, and Quang [15]. After extraction, the samples were centrifuged to remove insoluble material, and the supernatants were collected for further analysis. Protein quantification (2.6) as performed on the clarified extracts, which were subsequently sterilized by filtration through a 0.2 μm membrane filter and stored at controlled temperature until use in biological assays.

### *In vitro* Digestion and Preparation of the Digestion Product (PDV)

The *in vitro* digestion of *A. platensis* biomass was conducted to simulate physiological conditions of the human gastrointestinal tract. Freeze-dried biomass was subjected to a sequential digestion protocol involving oral, gastric, and intestinal phases. Initially, the biomass was incubated with a saliva solution (1 M) at 37 °C for 5 min under agitation. Subsequently, a gastric digestion step was performed by adding a simulated gastric solution (1 M) containing pepsin, adjusted to pH 3.0, followed by incubation for 2 h at 37 °C with continuous agitation. The intestinal phase was then initiated by the addition of simulated duodenal solution (1 M), simulated pancreatic solution containing trypsin and chymotrypsin, simulated bile solution (1 M), and a bicarbonate solution (1 M), with further incubation for 2 h at 37 °C under agitation. After completion of the digestion process, the resulting mixture was centrifuged to separate insoluble residues. The supernatant obtained corresponded to the *in vitro* digestion product (PDV), which was collected, sterilized by membrane filtration when necessary, and stored under appropriate conditions until use in antiparasitic assays.

### Obtaining *Giardiaduodenalis* Cysts

Stool samples containing *Giardia duodenalis* cysts were obtained from patients treated at the Oswaldo Cruz University Hospital (HUOC), located in Recife, Pernambuco (Brazil). Sample collection and handling were conducted in accordance with ethical standards and approved by the Research Ethics Committee of the Amaury de Medeiros Integrated Health Center – CISAM/UPE (CAAE: 30184720.6.0000.5191).

### Cysts Purification using an Aqueous Two-Phase System (PEG/Phosphate)

Cyst purification was carried out using an aqueous two-phase system (ATPS) composed of polyethylene glycol (PEG) and phosphate salts, following the methodology described by Amaral et al. [16]. PEG with a molecular weight of 4000 g/mol was used, and the phosphate phase was prepared using monosodium phosphate (NaH_2_PO_4_) and disodium phosphate (Na_2_HPO_4_). The ATPS was formulated with 10% (w/w) PEG 4000, 40% (w/w) phosphate salt solution, and 10% (w/w) diluted fecal sample, resulting in a total system mass of 2 g. All components were accurately weighed, transferred to graduated microtubes, and homogenized using an orbital shaker. The system was then allowed to stand at room temperature for 40 min to promote phase separation and equilibrium. After separation, aliquots from both phases were carefully collected using sterile syringes and needles for subsequent analyses. Cyst viability was evaluated using 0.1% eosin staining, followed by optical microscopy observation. It is important to note that this approach does not directly evaluate metabolic activity but serves as an indirect indicator of cellular damage.

### Protein Quantification

To standardize the different concentrations of extracts obtained by different techniques, protein concentration in aqueous extracts and PDV samples was determined using the bicinchoninic acid (BCA) protein assay kit (BCA™ Protein Assay Kit, Thermo Scientific), according to the protocol described by Smith et al. [17]. A standard calibration curve was constructed using bovine serum albumin (BSA) as the reference protein.

### Antiparasitic Activity Against *Giardiaduodenalis* Cysts

The antiparasitic activity of *A. platensis* aqueous extracts and the PDV was evaluated following the methodology described by Edlind et al. [18], with minor modifications. Purified viable cysts were quantified using a Neubauer chamber, and a suspension containing 1.5 × 10⁵ cysts was inoculated into each well of a 96-well microplate containing TYI-S-33 medium. Samples were treated with extracts or PDV at final concentrations of 15.6, 31.5, 62.5, 125, 250, and 500 µg/mL and incubated for 24 h at 37° C. Untreated cysts were used as negative controls, while cysts treated with 1% metronidazole served as positive controls. All assays were performed in triplicate and repeated in two independent experiments. Antiparasitic activity was determined based on cyst viability relative to control groups.

### Statistical Analysis

Half-maximal inhibitory concentration (IC_50_) values were calculated using GraphPad Prism version 5 (GraphPad Software Inc., San Diego, CA, USA). Statistical analyses were performed using one-way analysis of variance (ANOVA), followed by Dunnett’s post hoc test. Differences were considered statistically significant when *p* < 0.05.

## Results

### Purification of Cysts using an Aqueous Two-Phase System (PEG/phosphate)

The purification of *Giardia duodenalis* cysts is a critical step to ensure the reliability of antiparasitic assays, as it minimizes fecal debris and improves parasite visualization. The polyethylene glycol/phosphate aqueous two-phase system (PEG/phosphate ATPS) enabled efficient separation of cysts from fecal residues by exploiting differences in surface charge, density, and hydrophilic–hydrophobic interactions.

Cysts showed a preferential tendency to partition toward the PEG-rich phase; however, a considerable number of cysts were detected in both phases of the system. For subsequent analyses, the salt-rich phase was selected due to its lower viscosity and superior optical clarity, which facilitated staining procedures and microscopic observation, although it does not provide direct information on metabolic viability. Overall, the ATPS proved to be a rapid, simple, and cost-effective strategy for cyst purification, yielding samples with reduced background interference and suitable quality for downstream antiparasitic activity assays.

### Preliminary Screening Activity of *Arthrospiraplatensis* Aqueous Extracts Against *Giardiaduodenalis* Cysts

Prior to antiparasitic evaluation, the protein content of *A. platensis* aqueous extracts obtained by different extraction methods was determined. Protein concentrations of 2 mg/mL and 5 mg/mL were measured for extracts obtained by magnetic stirring and sonication, respectively. Both extracts exhibited inhibitory effects on *G. duodenalis* cyst viability in a concentration-dependent manner.

**Fig. 1:**
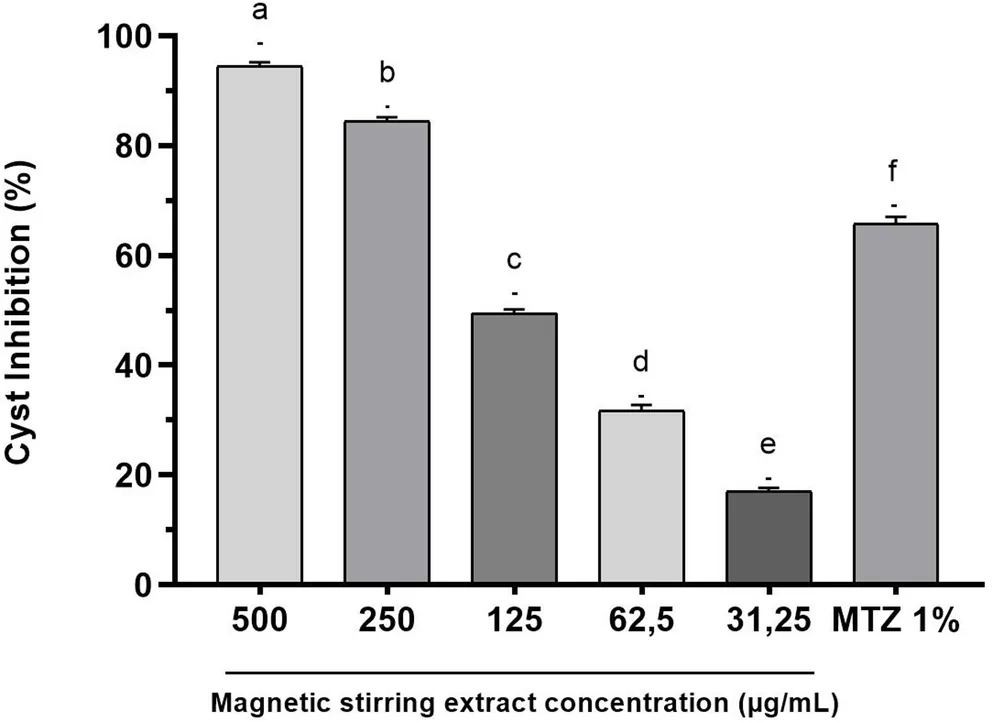
Minimum concentration of the magnetic stirring *A. platensis* extract to inhibit 50% of *Giardia duodenalis* cysts. Concentrations with letters that differ from each other are considered statistically significant (*p* < 0.05). MTZ: Metronidazole

For the extract obtained by magnetic stirring, inhibition rates of 95% and 85% were observed at concentrations of 500 µg/mL and 250 µg/mL, respectively, with an IC_50_ value of 153.1 µg/mL (Fig. 1). These inhibition rates were higher than those observed for the positive control treated with metronidazole, which exhibited 67% inhibition under the same experimental conditions. Microscopic analysis revealed the presence of non-viable trophozoites at extract concentrations of 62.5 µg/mL and 125 µg/mL. A progressive increase in cyst inhibition was observed with increasing extract concentrations, confirming a dose-dependent antiparasitic effect.

**Fig. 2:**
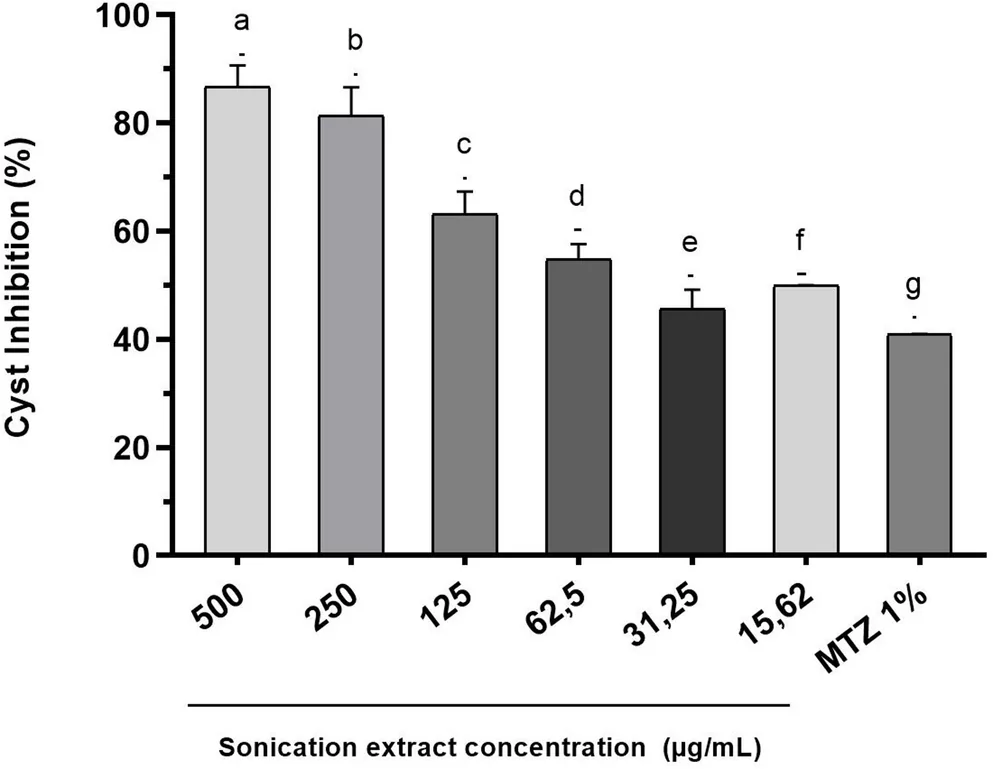
Minimum concentration of sonicated *A. platensis* extract to inhibit 50% of *Giardia duodenalis* cysts. Concentrations with letters that differ from each other are considered statistically significant (*p* < 0.05). MTZ: Metronidazole

The sonicated *A. platensis* extract also demonstrated pronounced inhibitory activity against *G. duodenalis* cysts. At concentrations of 500 µg/mL and 250 µg/mL, inhibition rates of 86.33% and 81.81% were recorded, respectively, which were significantly higher than those achieved with metronidazole (40%) (*p* < 0.05). The IC_50_ value for the sonicated extract was 95.4 µg/mL (Fig. 2), indicating greater potency compared to the extract obtained by magnetic stirring. Overall, the sonicated extract exhibited higher protein content and lower IC_50_ values, which correlated with enhanced antiparasitic activity against *G. duodenalis* cysts.

### Preliminary Screening Activity of the *in vitro* Digestion Product (PDV) of *Arthrospiraplatensis*

**Fig. 3:**
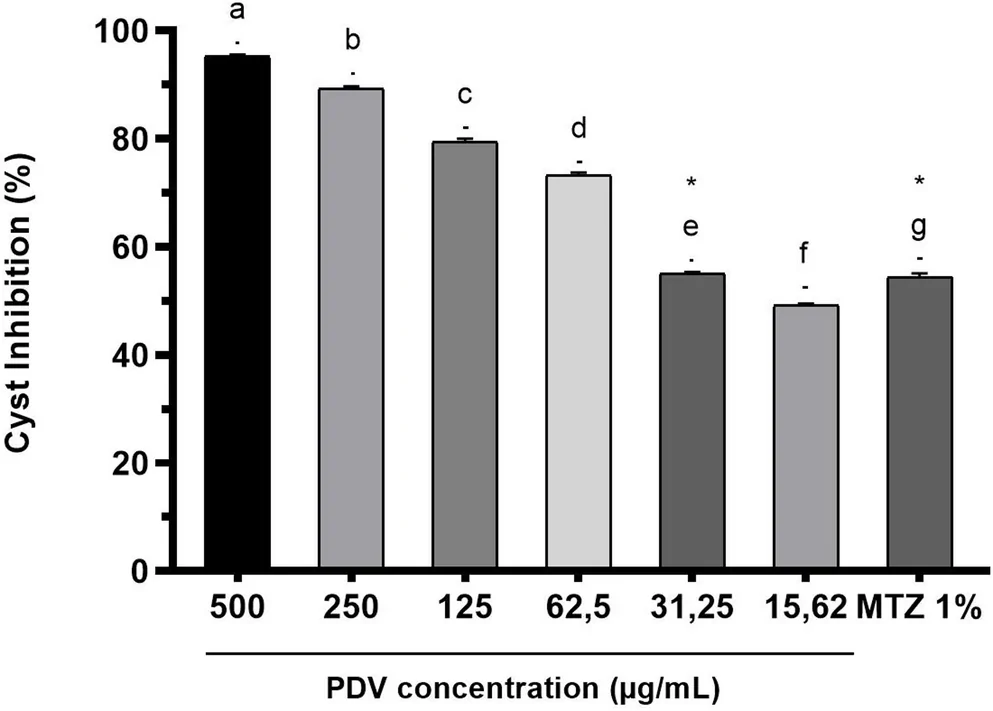
*In vitro* giardicidal activity of the protein digestion products (PDV) obtained from *Arthrospira platensis* against *Giardia duodenalis* cysts at different concentrations. Concentrations identified by different letters are significantly different from each other (*p* < 0.05). MTZ: Metronidazole

The *in vitro* digestion product (PDV) obtained from *A. platensis* biomass was evaluated for its antiparasitic activity against purified *G. duodenalis* cysts. The PDV contained proteins at concentrations ranging from 31.25 µg/mL to 500 µg/mL in aqueous solution. Notably, the *in vitro* digestion product showed an IC_50_ value of 43.6 µg/mL, representing the most effective result among the three extracts. The PDV exhibited significant membrane damage detected by eosin exclusion, with inhibition rates varying between 59% and 95% (Fig. 3).

At higher concentrations, the PDV demonstrated superior antiparasitic efficacy compared to the reference drug metronidazole. While metronidazole reached a maximum inhibition rate of 40% under the same experimental conditions, the PDV showed membrane damage in 75% to 95% of *G. duodenalis* cysts at the highest concentrations evaluated. These results highlight the enhanced effectiveness of the PDV, particularly in comparison with conventional therapy.

## Discussion

One of the central contributions of this study was the application of an aqueous two-phase system (ATPS/PEG-phosphate) for the purification of *Giardia duodenalis* cysts prior to antiparasitic evaluation. The results demonstrate that ATPS enabled effective separation of cysts from fecal debris, improving sample clarity and facilitating microscopic analysis. This aligns with findings from previous work, where ATPS systems have been successfully applied for the purification of protozoan cysts and oocysts, minimizing background interference and enhancing downstream biological assays [19, 20, 21, 22]. Efficient purification is critical in parasitological studies because fecal contaminants and debris can obscure cyst morphology and influence viability assessments, leading to variability in bioassay outcomes. ATPS separation relies on differences in density, hydrophobicity, and charge distribution between phases, enabling selective partitioning of target particles such as cysts [19, 22]. The present work reinforces the utility of ATPS as a robust, scalable, and low-cost preprocessing tool in protozoan bioactivity research.

The choice of *Arthrospira platensis* in this study is based on its recognized potential as a source of bioactive compounds of biotechnological interest. Due to its composition, rich in proteins, essential amino acids, polyunsaturated fatty acids, and other metabolites, this cyanobacterium has been associated with different biological activities [23]. Recognized for its high nutritional value, the use of Spirulina is safe and nutritious, as it has received FDA (Food and Drug Administration) certification as “GRAS” (Generally Recognized as Safe), ensuring that its consumption as food does not present health risks [24]. Furthermore, cytotoxicity assays previously performed by our research group, in models associated with Chagas disease, indicates the safe use of the extract, and corroborates with the antiparasitic action evidenced in the present study [25]. Due to the high protein content of *Arthrospira platensis*, protein quantification was performed to standardize the concentrations tested in the antiparasitic assays. Although this parameter guided the comparison between samples, the activity against *Giardia duodenalis* cysts is related to the bioactive compounds released by the different extraction methods.

The antiparasitic activity observed for *A. platensis* aqueous extracts against *G. duodenalis* cysts adds to a growing body of evidence that photosynthetic microorganisms, especially cyanobacteria and microalgae, can serve as sources of biologically active antiparasitic molecules [7]. These compounds have shown promise in treating various diseases, including leishmaniasis and giardiasis, due to their ability to target multiple biological pathways [8, 26]. While eosin exclusion staining is a standard, economical method widely used in Giardia cyst research [27, 28], it primarily assesses membrane integrity rather than metabolic viability. Excystation assays and *in vivo* infectivity studies would provide definitive confirmation of metabolic lethality. Previous studies have demonstrated antiparasitic effects of plant and algal extracts against *Giardia* and other protozoa, though methods and target life stages vary widely. Numerous plant‑derived compounds have shown activity against *Giardia* in both trophozoite and cyst stages. *In vitro* screens of 14 Mexican medicinal plants identified several extracts that reduced trophozoite viability measured by MTT assay [29]. Andrographolide from *Andrographis paniculata* inhibited trophozoite growth with an IC_50_ of 4.99 µM, induced ROS generation, DNA damage and cell‑cycle arrest [30]. Podophyllotoxin‑type lignans from *Bursera fagaroides* were active against both trophozoites and cysts [31], while hydroalcoholic oregano (*Origanum vulgare*) extract markedly killed cysts *in vitro* [30]. *In vivo* studies with *Artemisia annua* confirmed giardicidal effects and added anti‑inflammatory benefits [33]. Systematic reviews underscore the broad efficacy of these phytochemicals across life stages but note variability in experimental designs and a need for standardized protocols [34].

Although some reported IC_50_ values for crude extracts in the literature fall within similar ranges to those observed here, direct quantitative comparisons should be interpreted cautiously due to methodological differences in assay conditions, cyst/trophozoite stages evaluated, and extract composition. For example, *Ageratum conyzoides* crude extracts exhibited IC_50_ values ≤ 100 µg/mL against *G. duodenalis* trophozoites *in vitro*, with ultrastructural damage observed in key organelles, emphasizing the potential of plant-derived compounds [35].

The differential bioactivity observed between extracts obtained by sonication and magnetic stirring is consistent with findings reported in the literature. Sonication is known to promote effective cell wall disruption, thereby enhancing the release of intracellular constituents, including proteins and secondary metabolites, which may directly contribute to the observed biological effects [36]. Similar trends have been described for other microalgal species, in which ultrasound-assisted extraction conditions significantly influence extract composition and, consequently, bioactivity profiles. Studies by Meregalli et al. [30] and Rios-Romero et al. [31]. demonstrate that ultrasound-assisted extraction (UAE) improves the recovery of bioactive compounds from microalgae and other biomass sources, often resulting in extracts with enhanced biological performance.

Beyond aqueous extract activity, the inclusion of results from the *in vitro* digestion product (PDV) underscores the importance of considering physiologically relevant bioactivation pathways. The PDV demonstrated potent giardicidal activity across multiple concentrations and outperformed metronidazole under comparable conditions. This suggests that digestive processing of microalgal biomass may enhance the availability or transformation of specific bioactive moieties with antiparasitic properties. Moreover, non-protein compounds naturally present in *Arthrospira platensis*, such as pigments, lipids, or other secondary metabolites, may remain stable or become more bioavailable during digestion, thereby contributing to the observed antiparasitic effects. While direct mechanistic data are still limited, in vitro digestion models have been increasingly used to predict bioaccessibility and functional activity of food-derived compounds under gastrointestinal conditions, supporting the translational relevance of PDV findings. *In vitro* digestion models simulate the oral, gastric and intestinal phases of human digestion, allowing researchers to quantify the fraction of a compound that becomes bioaccessible—that is, released from the food matrix and solubilized for potential absorption [37], *in vitro* digestion models have been widely used to estimate the bioaccessibility and functional activity of food-derived compounds under gastrointestinal conditions, demonstrating that physiological digestion often enhances the release and availability of bioactive molecules that might otherwise remain bound within complex matrices [38]. By reproducing physiological conditions, static and dynamic gastrointestinal simulations provide mechanistic insight into the release, transformation and stability of bioactive moieties, thereby linking laboratory findings to likely *in vivo* effects.

Targeting *G. duodenalis* cysts holds particular epidemiological relevance because cyst viability directly influences infection transmission, environmental persistence, and susceptibility to water treatment processes. Enhancing cyst inactivation through natural compounds may contribute to complementary strategies for giardiasis control, particularly in regions where conventional therapies are less effective or less accessible [39]. Moreover, the demonstration of activity against cyst stages expands the potential utility of these compounds beyond trophozoite-focused assays common in most natural product screens.

*In vivo* studies provide further support for the relevance of natural products in giardiasis management. In murine models, several plant-derived compounds have demonstrated significant antiparasitic activity, in some cases comparable to or enhancing the efficacy of conventional nitroimidazoles. For instance, black tea (*Camellia sinensis*) exhibited a dose-dependent giardicidal effect in infected Swiss mice, with complete parasite clearance achieved at higher concentrations, while metronidazole alone showed only partial efficacy. Notably, the combined treatment resulted in complete elimination of the parasite, suggesting a synergistic interaction between natural compounds and conventional drugs [40]. Similarly, *Licochalcone A*, a flavonoid-derived compound, has shown potent anti-Giardia activity in both *in vitro* and *in vivo* models, highlighting its potential as a promising alternative to nitroimidazole-based therapies, particularly in the context of increasing drug resistance [41]. In addition, extracts from *Matricaria recutita* have demonstrated therapeutic effects in experimental giardiasis, significantly reducing cyst and trophozoite loads and improving pathological and immunological parameters, especially when used in combination with standard treatment [42].

Although the extrapolation from *in vitro* to *in vivo* outcomes requires careful validation, these findings collectively support the concept that natural and microalgal-derived compounds warrant deeper investigation as supplements or alternatives to conventional antiparasitic drugs. Taken together, the current results demonstrate that *A. platensis* aqueous extracts and PDV not only exhibit biologically relevant giardicidal activity but also underscore the influence of processing methods on bioactive compound yield and efficacy. This highlights the value of integrating sustainable extraction and digestion-based activation strategies in the development pipeline for antiparasitic agents derived from photosynthetic microorganisms.

## Conclusion

This study demonstrates that *Arthrospira platensis* is a promising and sustainable source of giardicidal biocompounds, with bioactivity strongly influenced by extraction strategy and digestive bioactivation. The superior performance of the *in vitro* digestion product highlights the importance of physiologically relevant processing in enhancing antiparasitic efficacy. These findings support the potential of microalgal-derived compounds as complementary alternatives for giardiasis control and provide a foundation for future mechanistic and translational investigations.

## Data Availability

No datasets were generated or analysed during the current study.

## Abbreviations

HUOC: Oswaldo cruz university hospital
PEG: Polyethylene glycol
BSA: Bovine serum albumin
ATPS: Aqueous two-phase system
